# Developing a patient-centred tool for pain measurement and evaluation in autosomal dominant polycystic kidney disease

**DOI:** 10.1093/ckj/sfaa259

**Published:** 2021-02-08

**Authors:** Ragada El-Damanawi, Michael Lee, Tess Harris, Laura B Cowley, Ingrid Scholtes, Simon Bond, Richard N Sandford, Ian B Wilkinson, Niek F Casteleijn, Marie C Hogan, Fiona E Karet Frankl, Thomas F Hiemstra

**Affiliations:** Department of Medicine, Division of Experimental Medicine and Immunotherapeutics, University of Cambridge, Cambridge, UK; Cambridge Clinical Trials Unit, Cambridge, UK; Department of Medicine, Division of Anaesthesia, University of Cambridge, Cambridge, UK; PKD Charity, London, UK; Cambridge Clinical Trials Unit, Cambridge, UK; Patient Led Research Hub, Cambridge Clinical Trials Unit, Cambridge, UK; Department of Medicine, Division of Anaesthesia, University of Cambridge, Cambridge, UK; Cambridge Clinical Trials Unit, Cambridge, UK; Department of Medical Genetics, University of Cambridge, Cambridge, UK; Department of Medicine, Division of Experimental Medicine and Immunotherapeutics, University of Cambridge, Cambridge, UK; Cambridge Clinical Trials Unit, Cambridge, UK; Department of Nephrology, University of Groningen, Groningen, The Netherlands; Department of Urology, University of Groningen, Groningen, The Netherlands; Division of Nephrology and Hypertension, Mayo Clinic, Rochester, NY, USA; Department of Medical Genetics, University of Cambridge, Cambridge, UK; Department of Medicine, Division of Experimental Medicine and Immunotherapeutics, University of Cambridge, Cambridge, UK; Cambridge Clinical Trials Unit, Cambridge, UK

**Keywords:** ADPKD, analgesia, chronic pain, pain, patient-reported outcomes

## Abstract

**Background:**

Pain affects 60% of the autosomal dominant polycystic kidney disease (ADPKD) population. Despite being an early and debilitating symptom, it is poorly characterized and management is suboptimal. This study aimed to develop an ADPKD-specific pain assessment tool (APAT) to facilitate pain research.

**Methods:**

Following a systematic review of PATs used in ADPKD studies and against international recommendations for pain trials, our multi-disciplinary team of clinical experts and patients constructed an ADPKD-pain conceptual framework of key pain evaluation themes. We compiled a new APAT covering domains prioritized within our framework using components of questionnaires validated in other chronic pain disorders. The APAT was administered longitudinally within a randomized high-water intake trial (NCT02933268) to ascertain feasibility and provide pilot data on ADPKD pain.

**Results:**

Thirty-nine ADPKD participants with chronic kidney disease Stages 1–4 provided 129 APAT responses. Each participant completed a median of 3 (range 1–10) assessments. Respondents’ mean ± standard deviation age was 47 ± 13 years; 59% (23) were female; and 69% (27) had enlarged kidneys with median time from diagnosis 14.2 (interquartile range 7.0–25.9) years. Pain (52%) and associated analgesic use (29%) were common. Pain severity was associated with increasing age [odds ratio (OR) = 1.07, P = 0.009], female gender (OR = 4.34, P = 0.018), estimated glomerular filtration rate <60 mL/min/1.73 m^2^ (OR = 5.45, P = 0.021) and hypertension (OR = 12.11, P = 0.007), but not with kidney size (P = 0.23). The APAT achieved good internal consistency (Cronbach’s alpha coefficient = 0.91) and test–retest reliability (domain intra-class correlation coefficients ranging from 0.62 to 0.90).

**Conclusions:**

The APAT demonstrated good acceptability and reliability, and following further validation in a larger cohort could represent an invaluable tool for future ADPKD pain studies.

## Graphical Abstract

**Graphical Abstract:**
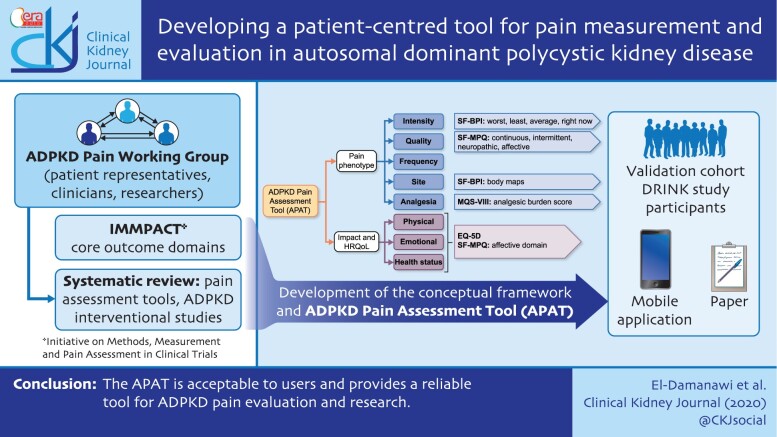

## INTRODUCTION

Chronic pain is one of the most debilitating features of autosomal dominant polycystic kidney disease (ADPKD) with an estimated prevalence of 60% [1]. The underlying mechanisms of ADPKD-related chronic pain (ACP) are complex and poorly understood. Cystic expansion resulting in traction on the kidney pedicle, capsule distension and compression of surrounding organs may all be contributing factors but provide incomplete explanation [2, 3]. Observational studies suggest that pain occurs early, often preceding kidney enlargement and functional decline by decades. There is no clear association between pain and kidney size until the kidneys become excessively enlarged [4, 5]. Pain may persist after resolution of an acute episode, suggesting development of aberrant sensory and autonomic pathways.

ACP is often refractory to standard treatments, with 39% of patients reporting inadequate analgesia [6]. Current pain management strategies follow a stepwise approach, with escalation through systemic analgesics and more invasive surgical options such as nerve blocks, cyst fenestration and nephrectomy [2, 3, 6–8]. Evidence for these interventions is limited and often extrapolated from generic pain trials [9–13]. More recently, tolvaptan has been shown to reduce acute kidney pain events [14].

Despite the potentially devastating impact of ACP on quality of life [5, 15, 16], clinicians place greater emphasis on clinical measures of disease progression. The Standardised Outcomes in Nephrology - Polycystic Kidney Disease (SONG-PKD) project seeks to establish standardized core outcomes for PKD trials through collaboration between key stakeholders [17] and has identified ACP as a core patient-reported outcome (PROM). However, the absence of validated ADPKD-specific pain assessment tools (APATs) is a major limitation. Disease-related pain is reported in only 22% of ADPKD clinical trials, with one report identifying 25 different PATs across 14 trials [18].

To date, there is only one ADPKD-specific PAT. The ADPKD Pain & Discomfort Scale (ADPKD-PDS) was developed by Otsuka Pharmaceutical Co. Ltd [19, 20], having identified three distinct pain patterns (chronic dull kidney pain, acute severe kidney pain, fullness/discomfort) from patient focus groups across Europe and the USA [21]. However, the ADPKD-PDS has important limitations including: (i) restriction to pain perceived to be from the kidneys (thus excluding components such related back pain); (ii) lack of validation in a representative ADPKD population; and (iii) lack of qualitative focus that may elucidate mechanisms. There is an urgent need for a validated, standardized patient-centred pain assessment to allow rigorous evaluation of pain management strategies and permit comparison between trials.

Classical assessment of pain requires a 3D approach that evaluates sensory–discriminative (intensity), affective–motivational (unpleasantness) and cognitive–evaluative (suffering) components [22–24]. The Initiative on Methods, Measurement and Pain Assessment in Clinical Trials (IMMPACT) consensus group outlined six core outcome domains for pain assessment in clinical trials focusing on PROM evaluating the nature of pain, impact on physical and emotional functioning and, where an intervention is administered, participant’s improvement rating, satisfaction and adverse events [25, 26].

We designed an APAT incorporating key pain dimensions and IMMPACT domains drawn from questionnaires validated in other pain conditions. Here, we report its use in a cohort of ADPKD patients with chronic kidney disease (CKD) Stages 1–4.

## MATERIALS AND METHODS

### Development of the conceptual framework and construction of the APAT

A working group consisting of ADPKD expert clinicians, pain specialists, researchers and patient representatives of the national UK PKD Charity, along with patient involvement leaders from the Cambridge Patient Led Research Hub, was convened and carried out a literature review to identify existing PAT in ADPKD research, evaluating their content and mapping these against the IMMPACT domains. The Cochrane Central Register of Controlled Trials and PubMed database were searched up to December 2019 with relevant search terms including ‘polycystic kidney disease’, ‘polycystic kidney disease, autosomal dominant’, ‘pain’, ‘pain assessment’ and ‘chronic pain’. Following screening of the titles and abstracts, we identified 676 studies; of these, 34 studies (8 observational and 26 interventional) were included in the qualitative synthesis (Supplementary Appendix S1, Figure S1). The IMMPACT and pain assessment domains were grouped as follows: (i) pain intensity; (ii) pain quality; (iii) temporal characteristics (frequency, prevalence and incidence); (iv) interference (emotional and physical functioning); and (v) analgesic burden. For interventional studies two additional measures were added: (vi) participant rating of improvement and treatment satisfaction and (vii) treatment-related adverse events. There was substantial heterogeneity in pain assessment, with 29 different tools across 34 studies.

**FIGURE 1:**
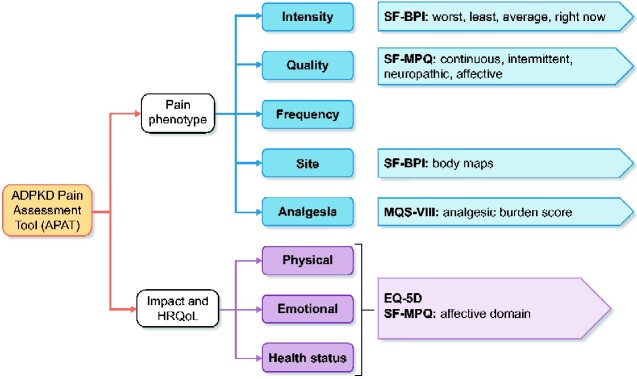
Conceptual framework identifying key themes for the APAT. HRQoL, health-related quality of life.

An ADPKD-pain-specific conceptual framework (Figure 1) outlining the key PAT themes was constructed, and generic pain questionnaires validated in other chronic pain diseases were reviewed to identify suitable questionnaires that captured these themes. An draft version of the APAT was created, refined through an iterative process and framed based on patient comprehension, ease of completion and ability to administer through several routes including a smartphone application developed for the DRINK (Determining feasibility of Randomisation to high vs ad libitum water Intake in Polycystic Kidney Disease) randomized trial [27, 28]. The smartphone application, available on iPhone operating system (iOS) and Android, was developed in collaboration with FatFractal Ltd [27]. The APAT was reviewed by a clinical experts group to ensure it captured the key concepts outlined by the framework, thus confirming face validity.

### Study design and participants

Clinical evaluation of the tool was performed by administering the APAT within the DRINK trial [27, 28]. DRINK was a prospective single-centre, open-label randomized controlled trial of high water (HW) versus *ad libitum* water intake in ADPKD patients with an estimated glomerular filtration rate (eGFR)  ≥20 mL/min/1.73 m^2^. Participants completed the APAT at baseline and Week 8, and were encouraged to complete the questionnaire more frequently if they experienced different types of pain to capture intra-individual pain experience variation.

### Description of APAT

The core elements of the APAT are outlined below (Supplementary Appendix SII).

#### EuroQol 5D scale—5 level

The EuroQol 5D (EQ-5D) scale is a standardized instrument for health status [29] and is the preferred UK measure of health-related quality of life [30]. It comprises five descriptive dimensions (mobility, self-care, usual activities, pain/discomfort and anxiety/depression) along with a visual analogue scale (VAS) for self-rated health status, and was also used to evaluate pain interference.

#### Modified Short-Form Brief Pain Inventory

The Modified Short-Form Brief Pain Inventory (SF-BPI) is a validated clinical PAT [31] that assesses pain severity and interference with affective (mood, sleep and enjoyment) and activity (walking and work) sub-dimensions. The questionnaire was used to assess the domain of pain intensity, including only the items contributing to the pain-intensity index scores. The index scores were pain at its (i) worst, (ii) least and (iii) average over a specified period (last 2 weeks), as well as (iv) now (current pain) using a numerical rating score (NRS) from 0 (no pain) to 10 (pain as bad as you can imagine). These items are validated as the Patient-Reported Outcomes Measurement Information System (PROMIS) pain-intensity short form [31, 32].

In the second part of the questionnaire, participants were directed to a body map where they were asked to indicate the site of (i) general, (ii) kidney-related and (iii) most severe pain using green, yellow and red shading on the smartphone application (or horizontal, vertical and X mark if on paper), respectively. Participants were able to freely use the body maps to indicate the location of any pains experienced across the whole body and to interpret which pains were kidney-related.

#### Modified Short-Form McGill Pain Questionnaire

The Modified Short-Form McGill Pain Questionnaire (SF-MPQ) [33] was designed to enable classification of pain symptomatology, with the potential to distinguish between neuropathic and non-neuropathic origins of pain. Pain quality is evaluated using 22 descriptors that are divided into four subclasses: (i) continuous pain descriptors (six items)—throbbing, cramping, gnawing, aching, heavy and tender; (ii) intermittent pain descriptors (six items)—shooting, stabbing, sharp, splitting, electric-shock and piercing; (iii) neuropathic (six items)—hot-burning, cold-freezing, light touch, itchy, pins and needles, and numbness; and (iv) affective descriptors (four items)—tiring-exhausting, sickening, fearful and punishing-cruel.

#### Pain frequency

Pain frequency over the preceding week was assessed using four categories ranging from 0–1 times/week to continuous.

#### Medication quantification scale version III

The Medication Quantification Scale (MQS) tool [34] is used to objectively quantify the medication regimen used in chronic pain populations. The MQS score for each medication is calculated using the medication class, dosage (sub-therapeutic, lower 50% of therapeutic dose, upper 50% of therapeutic dose and supra-therapeutic dose) and the agreed detriment/risk score, which was established prior to the US opioid crisis. The MQS scores for all medications are then summed to provide a total score for that individual, reflecting the analgesic burden.

### Data collection

Participants were invited to complete the questionnaire via the smartphone application or on paper. Smartphone application users were provided with a unique secure login, and encrypted data was securely transferred to a database hosted on an National Health Service (NHS) N3 server (Supplementary Appendix III).

### Data analysis

All participants submitting at least one questionnaire were included. For the primary analysis, data from both trial arms were combined. The potential impact of the High Water (HW) intervention on pain was explored by regression analyses. NRSs were either further sub-classified into groups or reported as frequencies and percentages, or appropriate summary statistics.

Cronbach’s internal consistency alpha coefficient and the average inter-item correlation score were determined to evaluate the reliability of the domain scores and explore whether any items did not fit the scale. For each domain, all numerical scores were first standardized by conversion to an 11-point scale (0–10) and reversed if necessary, such that zero indicated best health status or no problem, while 10 indicated worst health status or worst affected.

Mixed-effects logistic regression models were constructed, adjusting for covariates including age and gender, as well as measures of disease severity including hypertension, enlarged kidney size (kidney length≥16.5 cm or total kidney volume ≥750 mL/m^2^ on imaging), haematuria and CKD stage. Models were also adjusted for treatment group allocation and a time variant. The random intercept was unique subject identification to determine between-subject variances. The accepted level of statistical significance was indicated by an alpha <0.05. A subsequent analysis employing inter-class correlation coefficient was used to quantify test–retest reliability of pain severity index and other domains, assuming no effects of treatment allocation or study period.

### Study outcomes

The primary outcome of the study was to create a reliable APAT. Secondary outcomes included (i) to describe the ADPKD pain phenotype in our cohort and (ii) to determine the demographic and clinical characteristics that predict ACP and analgesic use.

## RESULTS

### Baseline characteristics

A total of 129 questionnaires were submitted by 93% (39/42) of enrolled trial participants (Table 1), with each completing a median of 3 [interquartile range (IQR) 2–7] APAT questionnaires. Of these, 79% (33/42) completed the questionnaire twice, 36% (15/42) completed it three times and 17% completed more then five questionnaires during the study. The mean ± standard deviation age of respondents was 47 ± 13 years, 90% (35) were of White British ethnicity and 59% (23) were female. The median (IQR) disease duration was 14.2 (7.0–25.9) years. Although the majority (69%, *n* = 27) had enlarged kidneys, only one participant had previous surgical intervention. Extra-renal manifestations were common, in particular hepatic cysts (59%, *n* = 23). Other predictors of disease progression including haematuria (28%) and hypertension (64%) were prevalent. The median (IQR) eGFR was 76 (47–111) mL/min/1.73 m^2^, with 38% having an eGFR <60 mL/min/1.73 m^2^.

Of the submitted questionnaires, 50% (64/129) had all elements completed. Pain frequency and severity were answered with the highest level of completeness, 100 and 89.9%, respectively. Lowest completion levels were found in the SF-MPQ (51% complete), body maps (51% complete) and EQ-5D (37% complete) sections. The majority (87.6%, 113/129) of questionnaires were submitted through the smartphone application.

### Pain severity—SF-BPI score

**FIGURE 2:**
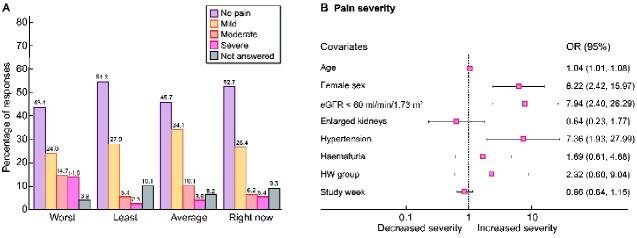
(**A**) BPI pain severity score for each of the four domains; worst pain, least pain and average pain over the last 2 weeks and pain right now (current pain), numbers on top of bars indicate percentage. (**B**) Demographic and clinical predictors of average BPI pain severity score. Age was centred around the mean. *X*-axis represents the OR. Advancing age, female sex, CKD Stage 3 or worse, and hypertension predicted increased pain severity.

Of all 129 responses, 45.7% indicated on average they had experienced no pain over the last 2 weeks, a further 34.1% had experienced mild pain (Scores 1–3), 10.1% had moderate pain (Scores 4–6) and 3.9% had severe pain (Score ≥7). In the worst pain item, 43.4% had no pain, 24.1% had mild, 14.7% had moderate and a further 14.0% indicated severe pain (Figure 2A).

In a multi-level multivariable ordered logistic regression model, age [odds ratio (OR) = 1.04, 95% confidence interval (CI) 1.01–1.08; P = 0.02], female gender (OR = 6.22, 95% CI 2.42–15.97; P = 0.000), CKD Stage 3 or worse (OR = 7.94, 95% CI 2.40–26.29; P = 0.001) and hypertension (OR = 7.36, 95% CI 1.93–27.99; P = 0.003) predicted higher pain severity scores, with no demonstrable association with kidney size, haematuria, treatment group allocation or change over time (Figure 2B). There was significant between-subject variability (OR = 6.92, 95% CI 2.89–16.6).

### Pain quality—SF-MPQ

**FIGURE 3:**
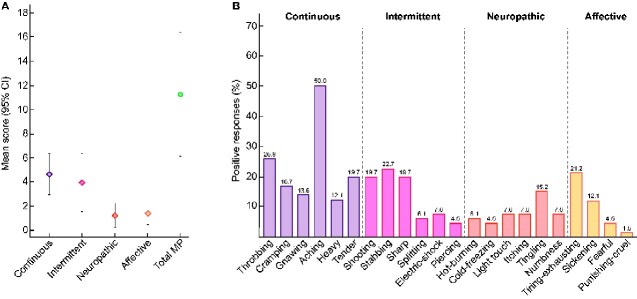
(**A**) Total mean score with 95% CIs for each of the MPQ descriptor subscales including the continuous, intermittent, neuropathic and affective scales. The final column represents the mean for all 22 descriptors. (**B**) The proportion of positive responses for each descriptor within the continuous, intermittent, neuropathic and affective pain domains.

Each of the 22 pain descriptors was scored using an 11-point NRS (0–10). The mean sum (95% CI) of all descriptors within the continuous subscale was 5.0 (95% CI 3.2–6.9), compared with 4.0 (95% CI 1.6–6.4) in the intermittent, 1.2 (95% CI 0.2–2.2) in the neuropathic and 1.4 (95% CI 0.5–2.3) in the affective subscales (Figure 3A). The overall total mean SF-MPQ score was 11.3 (95% CI 6.2–16.4), with a significant floor effect (defined as a score of zero) in the neuropathic (79.7%) and affective (76.6%) domains. In order to further differentiate the pain descriptors specifically used for ADPKD, each descriptor was categorized into description negative for participant (Score = 0) or pain description positive (Score ≥1). The adjectives reported with the highest frequency were aching (50%), throbbing (25.8%), tender (19.7%), stabbing (22.7%), shooting (19.7%), sharp (19.7%) and tiring-exhausting (21.2%). Neuropathic pain descriptors were not commonly used (Figure 3B).

### Pain frequency

Some 10.1% (13/129) of responses indicated pain frequency between 0 and 1 times over the last week; a further 81.4% (105/129) indicated a frequency of 2–3 times, 3.1% (4/129) experienced pain 4–5 times weekly and the remaining and 5.4% had pain that was either continuous or occurred ≥6 times in the preceding week.

### Site of pain

**FIGURE 4:**
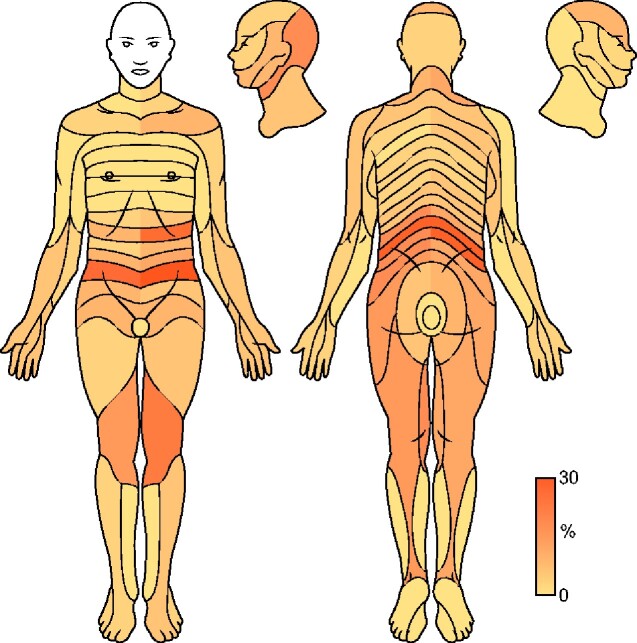
Heatmap indicating frequency (%) of pain sites affected by myotome. The body map is the same as that used by respondents in the APAT. Head and neck, lower abdomen, left upper quadrant, lumbo-sacral and radicular pain were most frequently indicated by participants.

Although participants were asked to indicate three types of pain (all, kidney-related and most severe) separately on the body maps, the majority (74%) only indicated ‘all’; therefore, data from all three were analysed collectively as the sites of all pain (Figure 4). Sites of the most frequent pain were lumbar-sacral and lower abdomen, left upper quadrant, followed by the posterior head and neck region, as well as pain affecting the thighs and knees.

### Analgesic medications—MQS III

**FIGURE 5:**
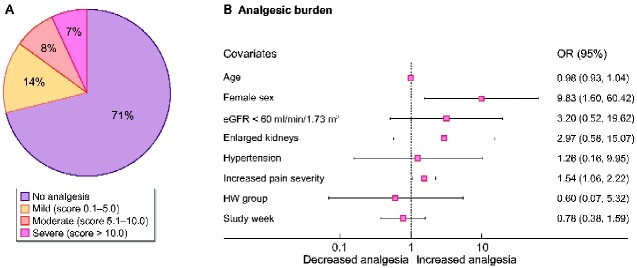
(**A**) Percentage of respondents in each category of analgesic burden; no analgesia, mild, moderate and severe analgesic burden. (**B**) Baseline predictors of increased analgesic burden, *X*-axis represents the OR, female sex and pain severity predict greater analgesic use.

Analgesic use was quantified by sub-classifying the MQS III score into four groups: (i) score = 0 (no analgesic burden); (ii) score = 0.1–5.0 mild; (iii) score = 5.1–10.0 moderate; and (iv) score >10 severe. Analgesia use was only collected at baseline and Week 8, with 72 responses available for analysis. Overall, 29.2% (21/72) indicated analgesic use during the study. Of these, 13.9% (10/72) had a mild analgesic burden, and a further 15.3% (11/72) had a moderate–severe burden (Figure 5A).

At baseline, 10.3% (4/39) used gabapentinoids, 2.6% (1/39) used non-steroidal anti-inflammatory drugs, 7.7% (3/39) used opiates, 12.8% (5/39) used triptans and 5.1% (2/39) were taking paracetamol.

Female gender (OR = 9.83, 95% CI 1.60–60.42; P = 0.014) and increased pain severity (OR = 1.54, 95% CI 1.06–2.22; P = 0.022) predicted greater analgesic use, while other indicators of disease severity including hypertension, large kidney size and worsening kidney function did not. In addition, analgesic burden did not change over the study period or with HW allocation (Figure 5B).

### EQ-5D health status and quality of life

**FIGURE 6:**
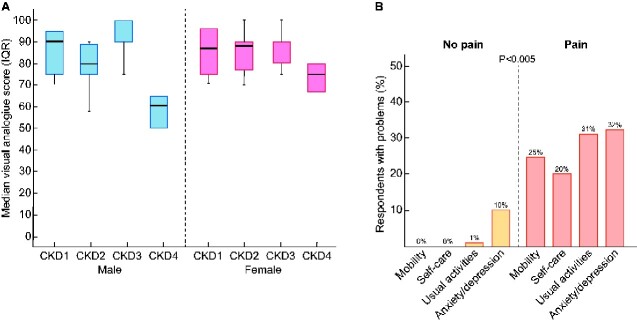
(**A**) Median VAS with hinges indicating IQR (25–75 centile) by CKD stage and gender. (B) EQ-5D health status domains, problems with mobility, self-care, usual activities as well as anxiety/depression were significantly more prevalent in those reporting pain.

The overall median (IQR) score for self-rated health status was 88 (75–93). Scores were similar for those with CKD Stage 1 [89 (IQR 79–95)], Stage 2 [85 (IQR 75–90)] and Stage 3 [90 (IQR 85–100)], although CKD Stage 4 was associated with a significantly reduced median (IQR) score of 63 (58–67) (P = 0.0001). This effect was more pronounced in males (Figure 6A). In a logistic regression model, the presence of advanced CKD and increased pain severity predicted lower VAS health scores, while hypertension predicted better scores (Table 2). Scores were unaffected by treatment group or study week. Those with a health status score of ≥75 had a younger age of hypertension onset compared with participants with lower scores (33.8; IQR 26.1–47.2 versus 39.4; IQR 32.7–50.5 years; P = 0.02).

**Table 2:** Mulitvaritate logistic regression model to determine predictors of VAS health scores

Parameter	OR (95% CI)	P-value
Age	1.03 (0.98–1.08)	0.24
Female gender	2.61 (0.95–7.23)	0.06
Advanced CKD stage	0.32 (0.17–0.61)	0.001
Large kidney size	0.66 (0.18–2.45)	0.53
Hypertension	7.25 (1.38–38.18)	0.02
Increased pain severity (SF-BPI)	0.68 (0.53–0.88)	0.003
Other comorbidities	0.92 (0.19–4.57)	0.92
HW group	0.46 (0.18–1.18)	0.11
Study week	0.98 (0.78–1.25)	0.90

Age was centred around the mean.

In the five health status domains, 81 responses were completed, 51.9% (42/81) reported having any problem with pain and discomfort. In the group reporting pain, problems with mobility (25% versus 0%; P = 0.000), self-care (20% versus 0%; P = 0.000) and usual activities (31% versus 1%; P = 0.000) were more prevalent compared with those not reporting any pain (Figure 6B). Participants with pain were also at higher risk of anxiety and depression [Relative risk (RR) = 2.97, 95% CI 1.70–5.20; P = 0.000].

Overall, participants completing more than the required two questionnaires (*n* = 15, 38%) had similar mean pain severity [1.4 (95% CI 0.4–2.5) versus 2.3 (95% CI 1.3–3.3); P = 0.24] and health status scores [80 (95% CI 67–94) versus 82 (95% CI 73–90); P = 0.84] compared with those completing two or fewer.

### Internal consistency

Cronbach’s alpha and the average inter-item correlation coefficients (AICCs) were calculated for the SF-BPI, SF-MPQ and EQ-5D domains (Table 3). The alpha coefficient exceeded the 0.70 reliability threshold for all domains. For the SF-BPI and SF-MPQ, the AICC and alpha coefficients remained stable with the removal of each item indicating that all items were well-fitting. Removal of the VAS health status score from the EQ-5D model led to a significant increase in the alpha coefficient and AICC, indicating that it did not strongly correlate with other items. The AICC for all the additive test scales exceeded the accepted range (0.15–0.50) indicating redundancy of some items. The percentage of patients reporting a floor effect ranged from 16% to 54%.

**Table 3:** APAT reliability testing using AICC and coefficient alpha for each item and the overall scale

Domain	Item	Mean score	95% CI	Ceiling (%)	Floor (%)	AICC	Coefficient alpha
SF-BPI^a^	Worst	2.5	2.0–3.0	2	43	0.83	0.93
	Least	1.0	0.7–1.3	0	54	0.81	0.93
	Average	1.4	1.1–1.8	0	46	0.76	0.91
	Current	1.3	0.9–1.7	0	53	0.78	0.91
Pain Severity Test scale						**0.79**	**0.94**
SF-MPQ^a^	Continuous	5.0	3.2–6.9	0	20	0.63	0.84
	Intermittent	4.0	1.6–6.4	0	31	0.64	0.84
	Neuropathic	1.2	0.2–2.2	0	40	0.66	0.85
	Affective	1.4	0.5–2.3	0	38	0.53	0.77
Pain Quality Test scale						**0.62**	**0.86**
EQ-5D^a^	Mobility	1.5	0.8–2.1	0	47	0.53	0.85
	Self-care	1.5	0.8–2.1	0	50	0.53	0.85
	Usual activities	1.6	1.0–2.2	0	43	0.53	0.85
	Pain and/or discomfort	1.9	1.3–2.4	0	30	0.56	0.87
	Anxiety and/or depression	1.8	1.2–2.4	0	36	0.54	0.86
	Health status	81	77–85	2	16	0.87	0.97
HRQoL test scale						**0.59**	**0.90**

a Although the NRS for each domain was standardized prior to inclusion in the model (as described in Materials and methods section), the original NRS for mean and CIs are displayed here. Values for the overall scales are in bold. HRQoL, health-related quality of life

### Test–retest reliability

An intra-class correlation (ICC) coefficient was determined for patients that provided a questionnaire at baseline and Week 8 (*n* = 33). The ICC coefficient for average pain severity, SF-MPQ and EQ-5D indicated moderate–excellent correlation for most domains (Table 4).

**Table 4:** ICC test for each of the main domains, indicating moderate to excellent correlation over time between baseline and Week 8 values

Domain	Item	Average ICC coefficient	95% CI	F-test with true value 0	
				Value	P-value
SF-BPI	Average severity	0.79	0.57–0.9	4.78	0.000
	Worst severity	0.73	0.45–0.87	3.72	0.000
	Least severity	0.87	0.73–0.94	7.52	0.000
	Current severity	0.88	0.74–0.94	8.76	0.000
SF-MPQ	Continuous	0.79	0.56–0.91	4.87	0.000
	Intermittent	0.9	0.78–0.95	9.92	0.000
	Neuropathic	0.83	0.63–0.92	5.83	0.000
	Affective	0.52	0.04–0.78	2.08	0.031
EQ-5D	Mobility	0.89	0.77–0.95	9.00	0.000
	Self-care	0.92	0.84–0.96	12.6	0.000
	Usual activities	0.76	0.49–0.88	4.08	0.000
	Pain/discomfort	0.78	0.55–0.9	4.63	0.000
	Anxiety/depression	0.85	0.68–0.93	6.56	0.000
	Health status	0.62	0.19–0.82	2.60	0.006

ICC values: <0.5 = poor, 0–5–0.75 = moderate, 0.75–0.9 = good and >0.9 = excellent correlation.

## DISCUSSION

We validated a bespoke PAT incorporating components from established chronic pain assessment instruments in an ADPKD cohort with CKD Stages 1–4. Our data show that ACP is prevalent and associated with an analgesic burden. Patients predominantly used agents higher up the analgesic ladder for which evidence in ADPKD was limited. Increasing age, female gender, along with traditional markers of disease severity but not large kidney size, were associated with pain severity, congruent with findings from previous studies [4, 5]. One possible explanation for the association of hypertension and pain severity is an inverse relationship between blood pressure (BP) and pain sensitivity, a phenomenon called BP-related hypoalgesia [35]. This effect is reversed in the presence of chronic pain, which subsequently predicts hypertension [36].

Uptake of the questionnaire was high, with responses predominantly via the smartphone application, allowing flexible completion and remote data collection. Pain frequency and severity achieved the highest levels of completion, while incompleteness in the remaining sections was partially explained by perceived relevance, lengthiness and mode of delivery. The larger number of items in the SF-MPQ and greater floor effect in the neuropathic and affective subclasses may have left respondents feeling overburdened with numerous descriptors. Furthermore, instructions for the body maps delivered via the application may have been confusing, requiring participants to record three different types of pain on a single map using a small mobile device screen. This will be addressed by providing three individual maps in future. Furthermore, the high values obtained for AICC indicate redundancy of some items, which could potentially be removed in future APAT iterations.

Self-reported health status scores were worse amongst those with greater pain severity and advanced CKD, and these effects were more pronounced in males. These variations may in part be explained by gender differences in health-seeking behaviours [37], as even though females reported greater pain and analgesic use compared with males, they still had better health scores. Paradoxically, hypertension was associated with higher self-reported health scores. One possible explanation is that the promotion of self-management through regular home BP monitoring in PKD outpatient clinics may have beneficial effects as demonstrated in other conditions [38].

Pain was predominantly chronic and non-neuropathic, suggesting that anti-neuropathic pain medications may be less effective in these phenotypes. Furthermore, chronicity coupled with the discrepancy between the prevalence of pain and analgesic use may indicate pain under-treatment or use of alternative non-pharmacological measures not captured in this study. Indeed, the use of self-medication and non-pharmacological interventions was prevalent in other studies [4, 39].

To date, there have been few studies describing the ACP phenotypes or clinical trials targeting pain in this cohort. Evidence is limited to case reports, observational data and small non-randomized studies, with greater focus on outcomes such as length of hospital stay and complication rates [12, 40–45]. Despite the increased reporting of pain in more recent PKD trials, current PATs are too heterogeneous to facilitate pain research [4, 14, 46–48]. Though the ADPKD-PDS [19, 20] has been rigorously developed, the primary focus on kidney-related pain is a challenge as previous evidence demonstrates that a minority of patients attribute pain to kidney disease [39], as supported by the APAT body maps. In addition, the APAT uses 22 pain descriptors, providing a much wider range of qualitative pain descriptions for patients to use. Therefore, by designing an APAT that allows broader capture of these pain syndromes, we are better positioned to understand the key influences of pain perceptions and experiences [23]. The APAT was also administered to ADPKD patients covering a spectrum of CKD Stages 1–4, although further validation through administration to larger cohorts is essential.

The APAT was developed with key stakeholders, with a pragmatic design to facilitate ease of completion and delivery in accessible formats, allowing the identification of several distinct ACP characteristics. It also included requisite components required by the IMMPACT consortium for the rigorous assessment of pain in randomized trials. Internal consistency was indicated by Cronbach’s alpha and the ICC was encouraging for reliability over time. Our study was limited by the modest sample size, and the characteristics of the patient cohort were limited to the inclusion criteria of the DRINK study and tended to include those less likely to be debilitated by pain compared with a typical ADPKD population. We did not collect data on self-management and non-pharmacological therapies. Furthermore, the APAT aim was to evaluate total pain burden in ADPKD; however, the contribution of other organomegaly to pain was not evaluated separately.

In this study, a reliable PAT was established. Future administration in larger cohorts will allow validation across a wider population, providing a useful standardized instrument for pain measurement in interventional PKD trials. Indeed, in the next stages, the APAT will be administered as part of a large observational ADPKD Pain study [Evaluating Chronic Pain in Autosomal Dominant Polycystic Kidney Disease using a Patient-Centred Approach to Data Collection and Synthesis: A National Prospective Observational Study (EASE-PKD)] recently funded by the National Institute for health research (NIHR). This will assist in the identification of ACP phenotypes more likely to achieve an analgesic treatment effects from novel interventions, allowing enrichment of trial populations and promoting greater precision medicine with targeted therapies.

## SUPPLEMENTARY DATA

Supplementary data are available at ckj online.

## Supplementary Material

sfaa259_Supplementary_Data
