# Supplementary material for: Developing a patient-centred tool for pain measurement and evaluation in autosomal dominant polycystic kidney disease
Source: Clin Kidney J. 2021 Feb 8;14(11):2338–48. doi: 10.1093/ckj/sfaa259 (PMC8573025; doi:10.1093/ckj/sfaa259)
Supplement: sfaa259_Supplementary_Data [file sfaa259_supplementary_data.zip › Appendix III_AppDr.pdf]

Appendix III - Smartphone Screenshots

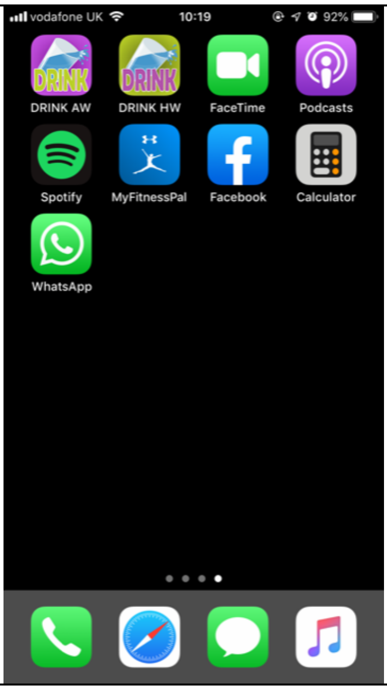

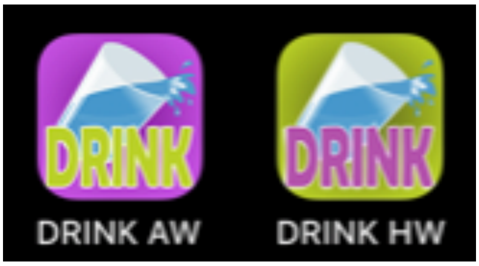

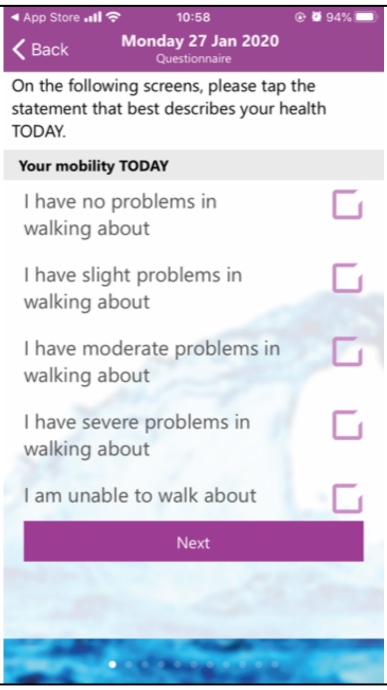

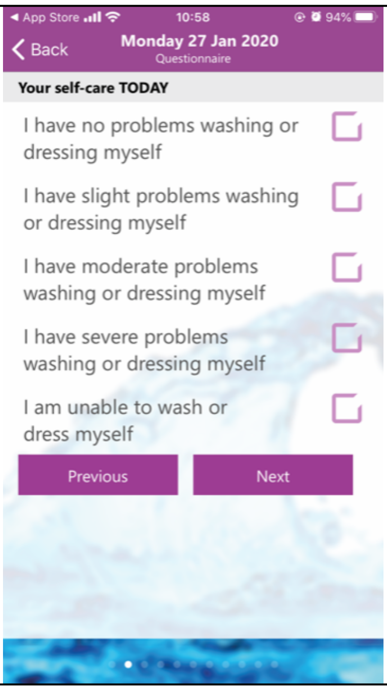

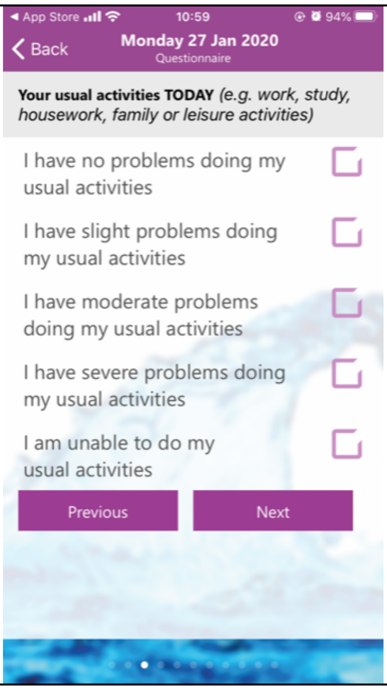
App Store 10:59 94%
Back
Monday 27 Jan 2020
Questionnaire
Your usual activities TODAY (e.g. work, study, housework, family or leisure activities)
I have no problems doing my usual activities
I have slight problems doing my usual activities
I have moderate problems doing my usual activities
I have severe problems doing my usual activities
I am unable to do my usual activities
Previous
Next

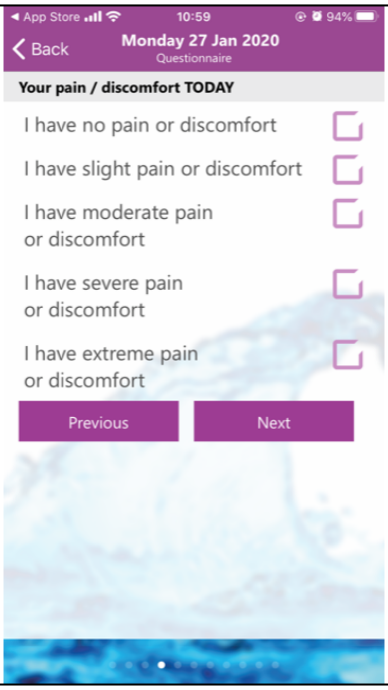
App Store 10:59 94%
Back
Monday 27 Jan 2020
Questionnaire
Your pain / discomfort TODAY
I have no pain or discomfort
I have slight pain or discomfort
I have moderate pain or discomfort
I have severe pain or discomfort
I have extreme pain or discomfort
Previous
Next

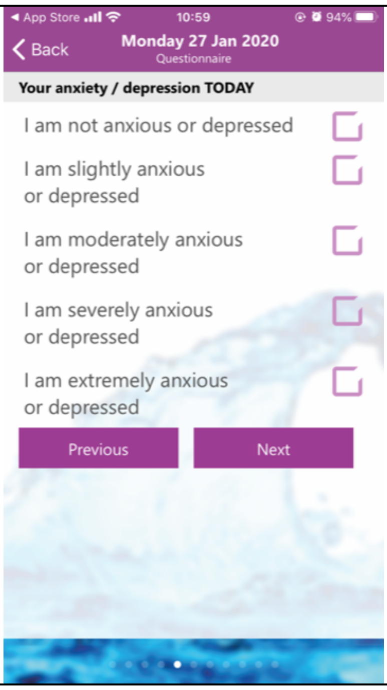
App Store 10:59 94%
Back
Monday 27 Jan 2020
Questionnaire
Your anxiety / depression TODAY
I am not anxious or depressed
I am slightly anxious or depressed
I am moderately anxious or depressed
I am severely anxious or depressed
I am extremely anxious or depressed
Previous
Next

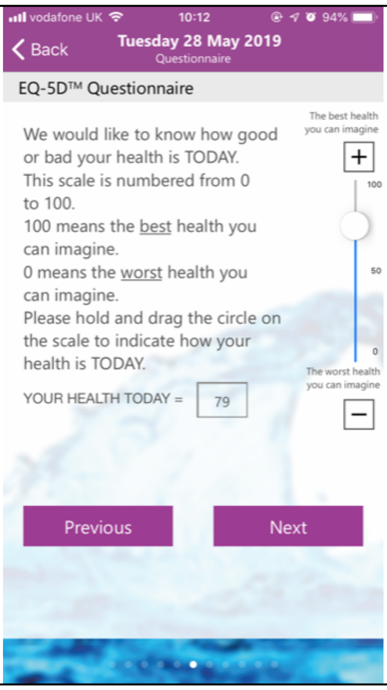
vodafone UK 10:12 94%
Back
Tuesday 28 May 2019
Questionnaire
EQ-5D™ Questionnaire
We would like to know how good or bad your health is TODAY.
This scale is numbered from 0 to 100.
100 means the best health you can imagine.
0 means the worst health you can imagine.
Please hold and drag the circle on the scale to indicate how your health is TODAY.
YOUR HEALTH TODAY = 79
The best health you can imagine
+
100
50
0
The worst health you can imagine
−
Previous
Next

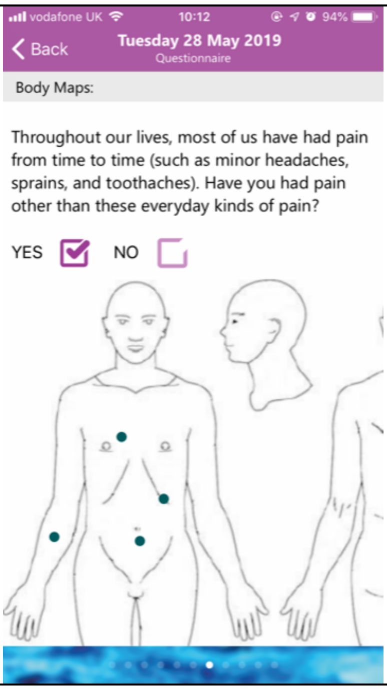
vodafone UK 10:12 94%
Back
Tuesday 28 May 2019
Questionnaire
Body Maps:
Throughout our lives, most of us have had pain from time to time (such as minor headaches, sprains, and toothaches). Have you had pain other than these everyday kinds of pain?
YES
NO

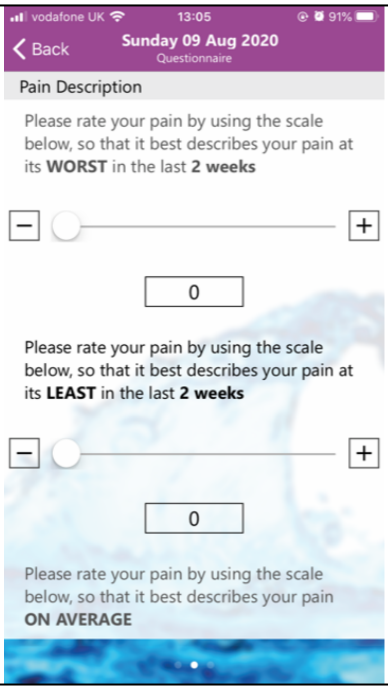
vodafone UK 13:05 91%
Back
Sunday 09 Aug 2020
Questionnaire
Pain Description
Please rate your pain by using the scale below, so that it best describes your pain at its WORST in the last 2 weeks
0
Please rate your pain by using the scale below, so that it best describes your pain at its LEAST in the last 2 weeks
0
Please rate your pain by using the scale below, so that it best describes your pain ON AVERAGE

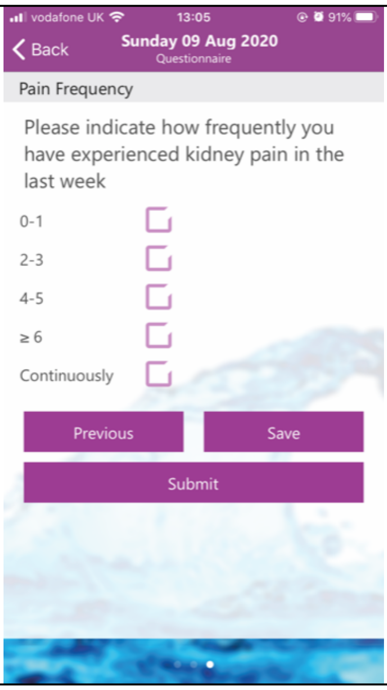
vodafone UK 13:05 91%
Back
Sunday 09 Aug 2020
Questionnaire
Pain Frequency
Please indicate how frequently you have experienced kidney pain in the last week
0-1
2-3
4-5
≥ 6
Continuously
Previous
Save
Submit

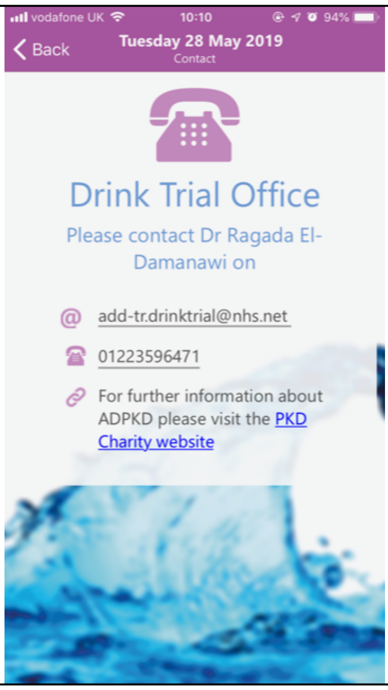
vodafone UK 10:10 94%
Back
Tuesday 28 May 2019
Contact
Drink Trial Office
Please contact Dr Ragada El-Damanawi on
add-tr.drinktrial@nhs.net
01223596471
For further information about ADPKD please visit the PKD Charity website
