# Supplementary material for: Developing a patient-centred tool for pain measurement and evaluation in autosomal dominant polycystic kidney disease
Source: Clin Kidney J. 2021 Feb 8;14(11):2338–48. doi: 10.1093/ckj/sfaa259 (PMC8573025; doi:10.1093/ckj/sfaa259)
Supplement: sfaa259_Supplementary_Data [file sfaa259_supplementary_data.zip › AppendixII_DRINKAPAT.pdf]

ADPKD Pain Assessment Tool (APAT)

Distribution of Pain

Throughout our lives, most of us have had pain from time to time (such as minor headaches, sprains, and toothaches). Have you had pain other than these everyday kinds of pains?

☐ Yes ☐ No

If yes, on the diagram;
Please shade using horizontal lines in the area where all your pain (s) are
Now shade using vertical lines where you feel your kidney problems are causing the pain
Put X on where pain hurts the most

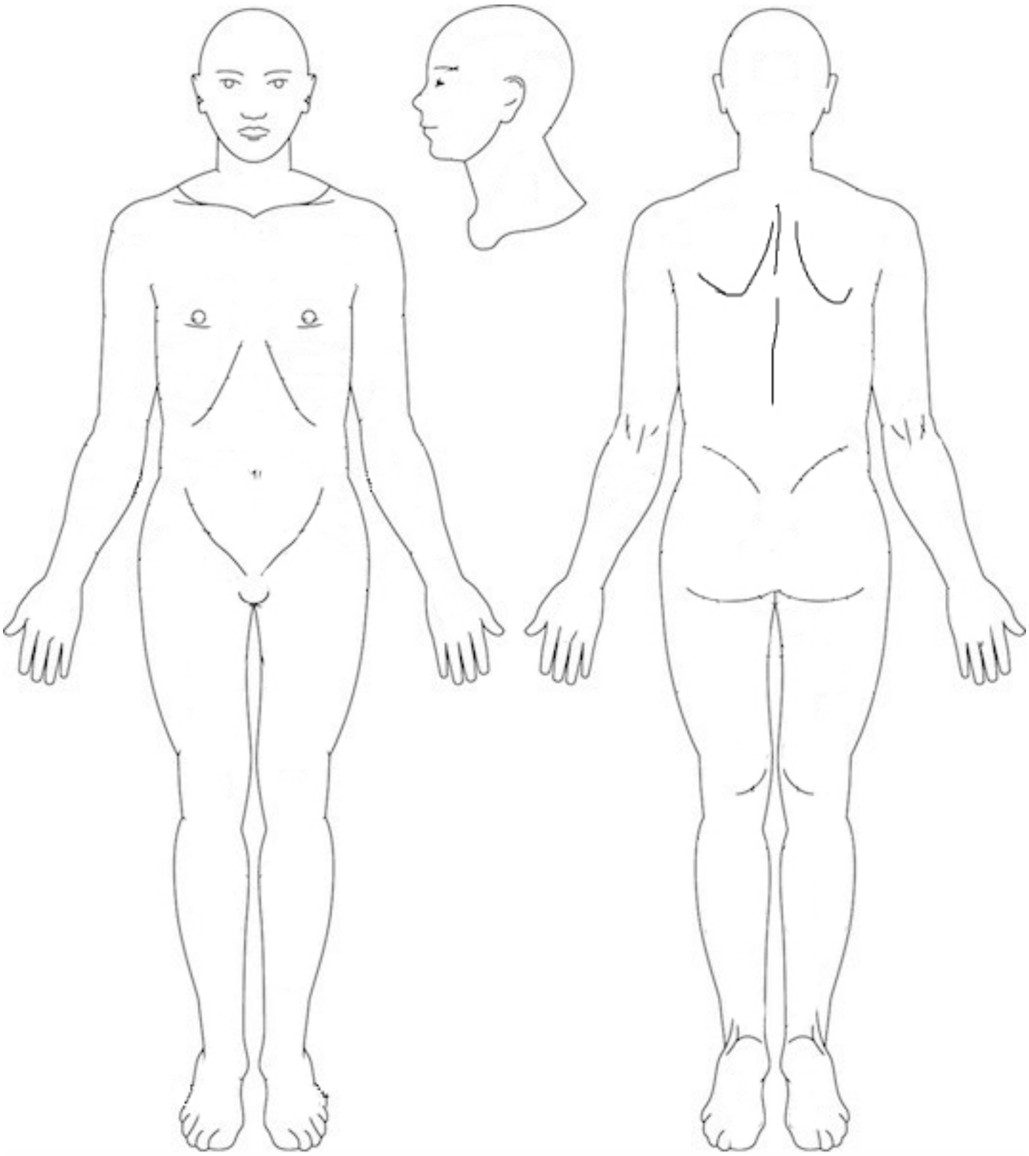

Pain Intensity

Please rate your pain by marking the box beside the number that best describes your pain at its **worst** in the last 2 weeks

☐0 ☐1 ☐2 ☐3 ☐4 ☐5 ☐6 ☐7 ☐8 ☐9 ☐10

No Pain — Pain as bad as you can imagine

Please rate your pain by marking the box beside the number that best describes your pain at its **least** in the last 2 weeks

☐0 ☐1 ☐2 ☐3 ☐4 ☐5 ☐6 ☐7 ☐8 ☐9 ☐10

No Pain — Pain as bad as you can imagine

Please rate your pain by marking the box beside the number that best describes your pain on the average

☐0 ☐1 ☐2 ☐3 ☐4 ☐5 ☐6 ☐7 ☐8 ☐9 ☐10

No Pain — Pain as bad as you can imagine

Please rate your pain by marking the box beside the number that tells how much pain you have right now

☐0 ☐1 ☐2 ☐3 ☐4 ☐5 ☐6 ☐7 ☐8 ☐9 ☐10

No Pain — Pain as bad as you can imagine

Pain Quality

**Throbbing Pain**	None	☐ 0	☐ 1	☐ 2	☐ 3	☐ 4	☐ 5	☐ 6	☐ 7	☐ 8	☐ 9	☐ 10	Worst Possible
**Shooting Pain**	None	☐ 0	☐ 1	☐ 2	☐ 3	☐ 4	☐ 5	☐ 6	☐ 7	☐ 8	☐ 9	☐ 10	Worst Possible
**Stabbing Pain**	None	☐ 0	☐ 1	☐ 2	☐ 3	☐ 4	☐ 5	☐ 6	☐ 7	☐ 8	☐ 9	☐ 10	Worst Possible
**Sharp Pain**	None	☐ 0	☐ 1	☐ 2	☐ 3	☐ 4	☐ 5	☐ 6	☐ 7	☐ 8	☐ 9	☐ 10	Worst Possible
**Cramping Pain**	None	☐ 0	☐ 1	☐ 2	☐ 3	☐ 4	☐ 5	☐ 6	☐ 7	☐ 8	☐ 9	☐ 10	Worst Possible
**Gnawing Pain**	None	☐ 0	☐ 1	☐ 2	☐ 3	☐ 4	☐ 5	☐ 6	☐ 7	☐ 8	☐ 9	☐ 10	Worst Possible
**Hot-burning Pain**	None	☐ 0	☐ 1	☐ 2	☐ 3	☐ 4	☐ 5	☐ 6	☐ 7	☐ 8	☐ 9	☐ 10	Worst Possible
**Aching Pain**	None	☐ 0	☐ 1	☐ 2	☐ 3	☐ 4	☐ 5	☐ 6	☐ 7	☐ 8	☐ 9	☐ 10	Worst Possible
**Tender**	None	☐ 0	☐ 1	☐ 2	☐ 3	☐ 4	☐ 5	☐ 6	☐ 7	☐ 8	☐ 9	☐ 10	Worst Possible
**Heavy Pain**	None	☐ 0	☐ 1	☐ 2	☐ 3	☐ 4	☐ 5	☐ 6	☐ 7	☐ 8	☐ 9	☐ 10	Worst Possible
**Splitting Pain**	None	☐ 0	☐ 1	☐ 2	☐ 3	☐ 4	☐ 5	☐ 6	☐ 7	☐ 8	☐ 9	☐ 10	Worst Possible
**Tiring-exhausting**	None	☐ 0	☐ 1	☐ 2	☐ 3	☐ 4	☐ 5	☐ 6	☐ 7	☐ 8	☐ 9	☐ 10	Worst Possible

**Sickening**	None	☐ 0	☐ 1	☐ 2	☐ 3	☐ 4	☐ 5	☐ 6	☐ 7	☐ 8	☐ 9	☐ 10	Worst Possible
**Fearful**	None	☐ 0	☐ 1	☐ 2	☐ 3	☐ 4	☐ 5	☐ 6	☐ 7	☐ 8	☐ 9	☐ 10	Worst Possible
**Punishing-cruel**	None	☐ 0	☐ 1	☐ 2	☐ 3	☐ 4	☐ 5	☐ 6	☐ 7	☐ 8	☐ 9	☐ 10	Worst Possible
**Electric-shock**	None	☐ 0	☐ 1	☐ 2	☐ 3	☐ 4	☐ 5	☐ 6	☐ 7	☐ 8	☐ 9	☐ 10	Worst Possible
**Cold-freezing**	None	☐ 0	☐ 1	☐ 2	☐ 3	☐ 4	☐ 5	☐ 6	☐ 7	☐ 8	☐ 9	☐ 10	Worst Possible
**Peircing**	None	☐ 0	☐ 1	☐ 2	☐ 3	☐ 4	☐ 5	☐ 6	☐ 7	☐ 8	☐ 9	☐ 10	Worst Possible
**Pain by Light-touch**	None	☐ 0	☐ 1	☐ 2	☐ 3	☐ 4	☐ 5	☐ 6	☐ 7	☐ 8	☐ 9	☐ 10	Worst Possible
**Itching**	None	☐ 0	☐ 1	☐ 2	☐ 3	☐ 4	☐ 5	☐ 6	☐ 7	☐ 8	☐ 9	☐ 10	Worst Possible
**Tingling or pins and needles**	None	☐ 0	☐ 1	☐ 2	☐ 3	☐ 4	☐ 5	☐ 6	☐ 7	☐ 8	☐ 9	☐ 10	Worst Possible
**Numbness**	None	☐ 0	☐ 1	☐ 2	☐ 3	☐ 4	☐ 5	☐ 6	☐ 7	☐ 8	☐ 9	☐ 10	Worst Possible

Pain Frequency

Please indicate how frequently you have experienced kidney pain in the last week?

- ☐ 0-1
- ☐ 2-3
- ☐ 3-4
- ☐ 5+
- ☐ Continuously

Analgesic Use

To be filled out by healthcare professional or member of the research team

Medication name	Dose	Frequency	Dosage score	Detriment	MQS Score
**Total MQS Score =**					
