# Supplementary material for: Developing a patient-centred tool for pain measurement and evaluation in autosomal dominant polycystic kidney disease
Source: Clin Kidney J. 2021 Feb 8;14(11):2338–48. doi: 10.1093/ckj/sfaa259 (PMC8573025; doi:10.1093/ckj/sfaa259)
Supplement: sfaa259_Supplementary_Data [file sfaa259_supplementary_data.zip › AppendixI_PainTools ADPKD Clinical Studies (2).docx]

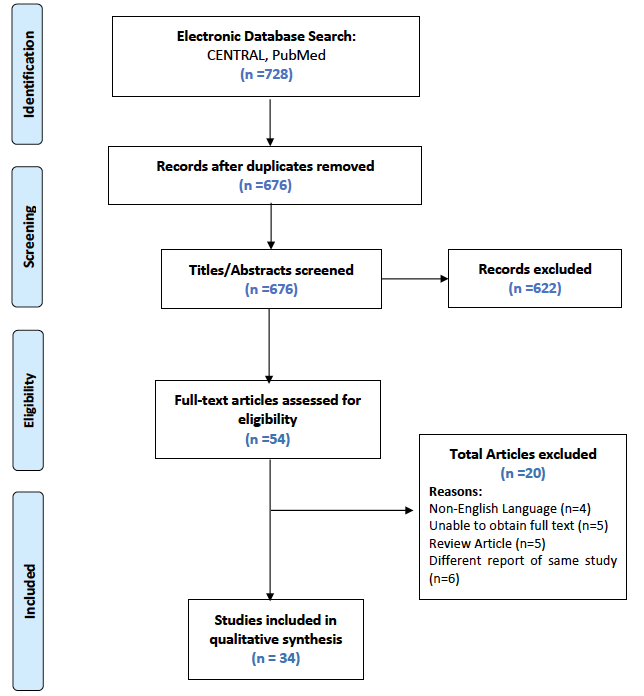


eFigure 1: PRISMA Diagram showing 676 unique studies were

found using the database search strategy. Of these 54 were

eligible for full text review, and 32 were included in the final

synthesis.

**Use of Pain Assessment Tools in ADPKD Clinical Studies**

| **Study** | **Trial Population** | **Design** | **Outcomes** | **Intervention** | **Pain Measurement tool** | **Domains Covered** |
| --- | --- | --- | --- | --- | --- | --- |
| **Arogundade et al 2018^1^** | N=41  Incident ADPKD patients over 18 years of age and fulfilling Ravine criteria | Prospective observational | Describe presentation and outcomes of ADPKD in Nigeria | Nil | Prevalence of pain | Temporal aspects |
| **Bajwa et al, 2004^2^** | N=171  Normal function to ESRD  Attending conference | Cross-sectional | Identify ADPKD pain patterns | Nil | 8-page questionnaire  Back, abdominal and chest pain, and headache  Pain nature (intensity, quality, site, frequency)  Medication history | Pain intensity  Pain quality  Temporal aspects  Analgesic burden |
| **Nishiura et al, 2013^3^** | N=97  ADPKD with preserved kidney function | Cross-sectional | Identify ADPKD pain patterns  (Brazil and Latin America) | Nil | Translated questionnaire used by Bajwa et al^2^ | Pain intensity  Pain quality  Temporal aspects  Analgesic burden |
| **Miskulin et al, 2014^4^** | N=1043  Hypertensive with baseline eGFR>20mls/min/1.73m2  (HALT-PKD study population at baseline) | Cross-sectional | Relationship between pain and disease severity | Nil | ﻿SF-36  Wisconsin Brief Pain Survey  Analgesic medication | Pain intensity  Pain quality  Interference  Temporal aspects  Analgesic burden |
| **Rizk et al, 2009**^5^ | N=152  Pre-dialysis ADPKD  ≥18 years old | Cross-sectional | Quality of life in ADPKD | Nil | SF-36  Physical component score  Mental component score  Pain medication (classified into predefined groups) | Pain Intensity  Interference  Analgesic burden |
| **Simms et al, 2015**^6^ | N=349  ADPKD  ≥18 years old | Cross-sectional | Quality of life in ADPKD | Nil | SF-36  Physical component score  Mental component score  Kidney disease specific domains included symptoms/problems, work status, cognitive function, quality of social interaction, sexual function, sleep  and social support and were scored (0–100) | Pain Intensity  Interference |
| **Suwabe et al, 2013**^7^ | N=219  ADPKD ≥ 20 years old | Cross-sectional | Quality of life in ADPKD | Nil | SF-36  Physical component score (PCS)  Mental component score (MCS)  Role/social component score (RCS)  Additional 12 ADPKD-specific questions | Pain Intensity  Interference |
| **Kim et al, 2019**^8^ | N= 364  Pre-RRT ADPKD | Prospective observational | Define ADPKD characteristics of Korean cohort | Nil | Prevalence of pain | Temporal aspects |
| - **Schrier et al, 2014 (HALT-PKD study A)**^9^ | N=588  15-49 years old  eGFR > 60ml/min/1.73m2 | Double blind, randomised, placebo-controlled trial  2x2 factorial | Primary: ΔTKV  Secondary: ΔeGFR, uACR, LVMI, pain frequency | ﻿Standard blood pressure target (120/70 to 130/80 mm Hg) vs a low target (95/60 to 110/75 mm Hg)    ACE inhibitor (lisinopril) plus an ARB (telmisartan) vs lisinopril plus placebo | <https://repository.niddk.nih.gov/media/studies/halt-pkd/Forms/F039%20Pain%20Questionnaire.pdf>  HALT-PKD Questionnaire | Pain intensity  Pain quality  Interference  Analgesic burden  Improvement  Adverse events |
| **Torres et al, 2014**  **(HALT-PKD study B)**^10^ | N=486  18-64 years old  eGFR 25-60ml/min/1.73m2 | Double blind, randomised, placebo-controlled trial | Primary composite:  time to death, end-stage renal disease, or a 50% reduction from the baseline eGFR  Secondary:  ΔuACR,  Δ Aldosterone  Incidence of pain | ACE inhibitor (lisinopril) and placebo vs lisinopril and an ARB (telmisartan) | <https://repository.niddk.nih.gov/media/studies/halt-pkd/Forms/F039%20Pain%20Questionnaire.pdf>  HALT-PKD Questionnaire | Pain intensity  Pain quality  Interference  Analgesic burden  Improvement  Adverse events |
| **Caroli et al 2013**^11^ | N= 79  ADPKD, ≥18 years old eGFR≥40ml/min/1.73m2 | Single blind, randomised, placebo-controlled trial | Primary outcomes:  TKV and total cyst volume  Secondary:  Kidney function decline  Adverse events | Octreotide LAR vs Placebo | Incidence of back pain and abdominal pain | Temporal aspects  Improvement  Adverse events |
| **Casteleijn et al, 2017**  **(TEMPO3:4)**^12^ | N=1445  18-50 years old  CrCl (C-G) ≥60 mL/min  TKV ≥750 mL | Double blind, randomised, placebo-controlled trial | Primary:  ΔTKV  Secondary:  Worsening kidney function (25% reduction in the reciprocal of the serum creatinine); clinically significant kidney pain, worsening hypertension and worsening albuminuria | Tolvaptan vs placebo | Incidence of acute kidney pain defined as events ﻿requiring medical intervention and that required documentation of clinical signs and symptoms that pain was kidney related (i.e. flank tenderness or evidence of cystic expansion or haemorrhage)  Subdivided in 5 categories according to pain severity and intervention used  Events | Temporal aspects  Improvement  Adverse events |
| **Casteleijn et al, 2017**^13^ | N=44  ﻿Pain present >3months; severity on a VAS score of ≥50 out of 100 | Non-randomised, single-centre, open-label | Primary:  ΔVAS score | Sequential nerve block and renal denervation | Pain intensity using VAS score (scale 0-100)  SF-36 | Pain Intensity  Interference  Analgesic burden  Improvement  Adverse events |
| **De Jager et al 2018**^14^ | N=5 ADPKD  kidney-related pain ≥3months with a visual analogue scale (VAS) score ≥50/100 | Non-randomised, single-centre, open-label | Outcomes  Pain  Analgesic use  Kidney function and blood pressure | Percutaneous renal denervation | Anatomical Therapeutic Chemical (ATC) classification system  McGill Pain Questionnaire (MPQ) - the validated Dutch questionnaire (MPQ-DV) | Pain Intensity  Pain Quality  Analgesic burden  Improvement  Adverse events |
| **Resnick et al, 2006**^15^ | N=4  Aged 15-19 years old  Chronic pain refractory to opioid analgesia | Non-randomised, single-centre, open-label | Outcomes:  Operative time, hospital stay, adverse events | Laparoscopic renal denervation | Bieri modified pain scale  Analgesia | Pain intensity  Analgesic burden  Improvement  Adverse events |
| **See et al, 2009**^16^ | N=21  ADPKD patients with cyst related pain, refractory to medical treatment, with no previous surgical intervention (included ESRD) | Non-randomised, single-centre, open-label | Primary:  Technical success rate of the procedure (collapsed cyst)  Secondary:  Creatinine, blood pressure, ESRD, symptom relief | Cyst ablation using N-bultyl cyanoacrylate (NBCA) and iodized oil | Relief of symptoms (abdominal distension and pain) | Pain intensity  Improvement  Adverse events |
| **Teichman et al, 1995^17^** | N=6  ADPKD with refractory cyst pain – failed previous percutaneous drainage | Non-randomised, single-centre, open-label | Outcomes:  Operative time, blood loss, length of hospital stay, pain relief | Laparoscopic marsupialisation | Pain relief | Pain intensity  Improvement  Adverse events |
| **Haseebuddin et al, 2012^18^** | N=37 | Non-randomised, single-centre, open-label | Outcomes:  Renal function  Hypertension  Pain control | Laparoscopic cyst decortication | Telephone pain questionnaire: preoperative and current opioid use, subjective pain relief and readiness to repeat surgery with full hindsight  Proportion reporting >50% pain relief | Pain intensity  Analgesic burden  Improvement  Adverse events |
| **McNally et al, 2001^19^** | N=7  ADPKD with ESRD and cyst related pain | Non-randomised, single-centre, open-label | Outcome:  Δ Symptoms | Laparoscopic cyst decortication | Standardised Pain scale (0-10) | Pain intensity  Improvement  Adverse events |
| **Lee et al, 2003^20^** | N=29  ADPKD patients with chronic pain, hypertension, renal insufficiency (CrCl) | Non-randomised, single-centre, open-label | Outcomes:  ΔPain scores  ΔHypertension  ΔKidney function | Laparoscopic cyst decortication | Pain Analogue scale  Relative pain relief (RPR) = (preoperative score - postoperative score) ÷ (preoperative pain score) | Pain intensity  Improvement  Adverse events |
| **Lifson et al, 1998^21^** | N=8  ADPKD with chronic pain | Non-randomised, single-centre, open-label | Outcomes:  Blood loss, length of hospital stay, complications | Laparoscopic cyst decortication | Pain free status | Pain intensity  Improvement  Adverse events |
| **Qian et al, 2015^22^** | N=70  ADPKD patients that underwent decortication | Retrospective observational | Outcomes:  Efficacy of decortication by ADPKD stage –  Intraoperative: recovery time, pain medication dose, hospital stay  Post-operative: change in serum creatinine at 1 and 3 years follow up | Open cyst decortication | Pain Medication dose  VAS (0-10) | Pain intensity  Analgesic burden  Improvement  Adverse events |
| **Lee et al, 2003^23^** | N=11  ADPKD with abdominal or flank pain | Non-randomised, single-centre, open-label | Outcomes:  Subjective pain relief | Cyst ablation with absolute ethanol | Proportion pain free at one year | Pain intensity  Improvement  Adverse events |
| **Elzinga et al, 1992^24^** | N=30  ADPKD with chronic pain and renal insufficiency (Cr >1.4mg/dl) | Non-randomised, single-centre, open-label | Outcomes:  Δ Kidney function (Cr, eGFR, DMSA)  ΔBlood pressure  Δ Symptoms | Surgical cyst decompression | Time to event – being pain free | Pain intensity  Improvement  Adverse events |
| **Asimakopoulos et al 2015**^25^ | N=19  End stage ADPKD, pre-transplant | Retrospective  single-centre | Outcomes:  Procedure success and duration  Blood loss  Hospital stay  Complications | Laparoscopic nephrectomy and morcellation | No pain data collected | Adverse events |
| **Binsaleh et al, 2006**^26^ | N=12  ADPKD requiring nephrectomy – indications variable – pain, pre-transplant, recurrent infection | Retrospective  single-centre | Outcomes:  Pre, intra and post-operative parameters | Laparoscopic versus open nephrectomies | Mean analgesic requirement (mg of morphine) | Analgesic burden  Improvement  Adverse events |
| **Lipke et al, 2007^27^** | N=18  Patients that had undergone bilateral hand assisted laparoscopic nephrectomy | Retrospective  single-centre | Outcomes:  Δ Pain  Safety | Bilateral hand assisted laparoscopic nephrectomy | VAS (0-10) | Pain intensity  Improvement  Adverse events |
| **Dunn et al, 2000^28^** | N=9  ADPKD with ESRD, chronic flank or abdominal pain | Non-randomised, single-centre, open-label | Outcomes:  Analgesic use, hospital course, and convalescence | Laparoscopic nephrectomy | Pain analogue score (0-10)  Quality of life  Analgesia | Pain intensity  Interference  Improvement  Analgesic burden  Adverse events |
| **Desai et al, 2008^29^** | N=13  ADPKD on haemodialysis, Pre-transplant laparoscopic nephrectomy | Retrospective  single-centre | Outcomes:  Operative duration, haemoglobin decrease, blood transfusion rate, hospital stay, analgesic requirement, and time to receipt of renal transplant | Laparoscopic nephrectomy | Analgesic requirement (mg of tramadol) | Improvement  Analgesic burden  Adverse events |
| **Sakuhara et al, 2015^30^** | N=15  ADPKD on long term dialysis, clinical symptoms due to enlarged kidneys | Non-randomised, single-centre, open-label | Outcomes:  Kidney volume reduction  Δ Symptoms | Trans-catheter arterial embolization (TAE) using ethanol | Change in symptom score for abdominal/lower back pain  0 (marked worsening) – 5 (no change) – 10 (marked improvement) | Pain intensity  Improvement  Adverse events |
| **Ubara et al, 2002^31^** | N=64  ﻿ADPKD on dialysis, anuric/oligoanuric, with compression related symptoms | Non-randomised, single-centre, open-label | Outcomes:  ﻿Renal size, abdominal circumference, dry weight, haematocrit, and insulin-like growth factor-I | ﻿Trans-catheter arterial embolization (TAE) using ethanol | Unknown – “subjective symptomatic relief” | Pain intensity  Improvement  Adverse events |
| **Suwabe et al, 2017^32^** | N=188  ADPKD on haemodialysis | Non-randomised, single-centre, open-label | Outcomes:  Change in SF-36 scores | Trans-catheter arterial embolization (TAE | SF-36  Physical component score (PCS)  Mental component score (MCS)  Role/social component score (RCS)  15-item questionnaire  that assessed specific symptoms of ADPKD | Pain Intensity  Interference  Improvement  Adverse events |
| **Elashry et al, 1996^33^** | N=4  (7 procedures)  ADPKD | Non-randomised, single-centre, case reports | Outcomes:  Operative time, blood loss, analgesia. Symptomatic relief | 5 laparoscopic cyst decortications  2 laparoscopic nephrectomies | Mean parenteral analgesia (mg of morphine)  Pain analogue score (0-10) | Pain intensity  Improvement  Analgesic burden  Adverse events |
| **Sulikowski et al, 2006^34^** | N=30  Pre-dialysis ADPKD | Non-randomised, single-centre, open-label | Outcomes:  Operative time, post-operative pain, length of hospital stay | Laparoscopic polycyst removal versus nephrectomy | VAS (0-10)  Analgesic use | Pain intensity  Improvement  Analgesic burden  Adverse events |

eTable 1: Studies Included in the qualitative synthesis. Pain assessment domains included in each study are outlined in the final column

**References:**

1. Arogundade, F. A. *et al.* Clinical presentation and outcome of autosomal dominant polycystic kidney disease in Nigeria. *Afr. Health Sci.* **18**, 671–680 (2018).

2. Bajwa, Z. H., Sial, K. A., Malik, A. B. & Steinman, T. I. Pain patterns in patients with polycystic kidney disease. *Kidney Int.* **66**, 1561–1569 (2004).

3. Nishiura, J. L., Eloi, S. R. M. & Heilberg, I. P. Pain determinants of pain in autosomal dominant polycystic kidney disease. *Jornal brasileiro de nefrologia : ’orgao oficial de Sociedades Brasileira e Latino-Americana de Nefrologia* **35**, 242–243 (2013).

4. Miskulin, D. C. *et al.* Health-Related Quality of Life in Patients With Autosomal Dominant Polycystic Kidney Disease and CKD Stages 1-4: A Cross-sectional Study. *Am. J. Kidney Dis.* **63**, 214–226 (2014).

5. Rizk, D. *et al.* Quality of Life in Autosomal Dominant Polycystic Kidney Disease Patients not yet on Dialysis. *Clin. J. Am. Soc. Nephrol.* **4**, 560–566 (2009).

6. Simms, R. J., Thong, K. M., Dworschak, G. C. & Ong, A. C. M. Increased psychosocial risk, depression and reduced quality of life living with autosomal dominant polycystic kidney disease. *Nephrol. Dial. Transplant.* **31**, 1130–1140 (2016).

7. Suwabe, T. *et al.* Quality of life of patients with ADPKD—Toranomon PKD QOL study: cross-sectional study. *BMC Nephrol.* **14**, 179 (2013).

8. Kim, H. *et al.* Baseline characteristics of the autosomal-dominant polycystic kidney disease sub-cohort of the KoreaN cohort study for outcomes in patients with chronic kidney disease. *Nephrology (Carlton).* **24**, 422–429 (2019).

9. Schrier, R. W. *et al.* Blood Pressure in Early Autosomal Dominant Polycystic Kidney Disease. *N. Engl. J. Med.* **371**, 2255–2266 (2014).

10. Torres, V. E. *et al.* Angiotensin Blockade in Late Autosomal Dominant Polycystic Kidney Disease. *N. Engl. J. Med.* **371**, 2267–2276 (2014).

11. Caroli, A. *et al.* Effect of longacting somatostatin analogue on kidney and cyst growth in autosomal dominant polycystic kidney disease (ALADIN): a randomised, placebo-controlled, multicentre trial. *Lancet* **382**, 1485–1495 (2013).

12. Casteleijn, N. F. *et al.* Tolvaptan and Kidney Pain in Patients With Autosomal Dominant Polycystic Kidney Disease: Secondary Analysis From a Randomized Controlled Trial. *Am. J. Kidney Dis.* **69**, 210–219 (2017).

13. Casteleijn, N. F. *et al.* Novel treatment protocol for ameliorating refractory, chronic pain in patients with autosomal dominant polycystic kidney disease. *Kidney Int.* **91**, 972–981 (2017).

14. de Jager, R. L. *et al.* Catheter-based renal denervation as therapy for chronic severe kidney-related pain. *Nephrol. Dial. Transplant* **33**, 614–619 (2018).

15. Resnick, M., Chang, A. Y. & Casale, P. Laparoscopic Renal Denervation and Nephropexy for Autosomal Dominant Polycystic Kidney Disease Related Pain in Adolescents. *J. Urol.* **175**, 2274–2276 (2006).

16. See, H. K., Seung, H. K. & Jeong, Y. C. Cyst ablation using a mixture of N-butyl cyanoacrylate and iodized oil in patients with autosomal dominant polycystic kidney disease: The long-term results. *Korean J. Radiol.* **10**, 377–383 (2009).

17. Teichman, J. M., Hulbert, J. C., H Teichman, J. M. & Hulbert, J. C. *Laparoscopic Marsupialization of the Painful Polycystic Kidney*. *The Journal of urology* **153**, (1995).

18. Haseebuddin, M. *et al.* Long-term impact of laparoscopic cyst decortication on renal function, hypertension and pain control in patients with autosomal dominant polycystic kidney disease. *J. Urol.* **188**, 1239–1244 (2012).

19. McNally, M. L., Erturk, E., Oleyourryk, G. & Schoeniger, L. Laparoscopic cyst decortication using the harmonic scalpel for symptomatic autosomal dominant polycystic kidney disease. *J. Endourol.* **15**, 597–9 (2001).

20. Lee, D. I. *et al.* Laparoscopic cyst decortication in autosomal dominant polycystic kidney disease: Impact on pain, hypertension, and renal function. *J. Endourol.* **17**, 345–354 (2003).

21. Lifson, B. J., Teichman, J. M. & Hulbert, J. C. Role and long-term results of laparoscopic decortication in solitary cystic and autosomal dominant polycystic kidney disease. *J. Urol.* **159**, 702–5; discussion 705-6 (1998).

22. Qian, X. *et al.* Which Stage of ADPKD Is More Appropriate for Decortication? A Retrospective Study of 137 Patients from a Single Clinic. *PLoS One* **10**, e0120696 (2015).

23. Lee, Y. R. & Lee, K.-B. Ablation of symptomatic cysts using absolute ethanol in 11 patients with autosomal-dominant polycystic kidney disease. *Korean J. Radiol.* **4**, 239–242 (2003).

24. Elzinga, L. W. *et al.* Cyst decompression surgery for autosomal dominant polycystic kidney disease. *J. Am. Soc. Nephrol.* **2**, 1219–26 (1992).

25. Asimakopoulos, A. D. *et al.* Laparoscopic pretransplant nephrectomy with morcellation in autosomic-dominant polycystic kidney disease patients with end-stage renal disease. *Surg. Endosc.* **29**, 236–244 (2015).

26. Binsaleh, S., Luke, P. P., Nguan, C. & Kapoor, A. Comparison of laparoscopic and open nephrectomy for adult polycystic kidney disease: operative challenges and technique. *Can. J. Urol.* **13**, 3340–5 (2006).

27. Lipke, M. C., Bargman, V., Milgrom, M. & Sundaram, C. P. Limitations of Laparoscopy for Bilateral Nephrectomy for Autosomal Dominant Polycystic Kidney Disease. *J. Urol.* **177**, 627–631 (2007).

28. Dunn, M. D. *et al.* Laparoscopic nephrectomy in patients with end-stage renal disease and autosomal dominant polycystic kidney disease. *Am. J. Kidney Dis.* **35**, 720–725 (2000).

29. Desai, M. R., Nandkishore, S. K., Ganpule, A. & Thimmegowda, M. Pretransplant laparoscopic nephrectomy in adult polycystic kidney disease: a single centre experience. *BJU Int.* **0**, 071008065132003-??? (2008).

30. Sakuhara, Y. *et al.* Transcatheter Arterial Embolization with Absolute Ethanol Injection for Enlarged Polycystic Kidneys after Failed Metallic Coil Embolization. *J Vasc Interv Radiol* **19**, 267–271 (2008).

31. Ubara, Y. *et al.* Renal contraction therapy for enlarged polycystic kidneys by transcatheter arterial embolization in hemodialysis patients. *Am. J. Kidney Dis.* **39**, 571–579 (2002).

32. Suwabe, T. *et al.* Effect of renal transcatheter arterial embolization on quality of life in patients with autosomal dominant polycystic kidney disease. *Nephrol. Dial. Transplant* **32**, 1176–1183 (2017).

33. Elashry, O. M., Nakada, S. Y., Wolf, J. S. J., McDougall, E. M. & Clayman, R. V. Laparoscopy for adult polycystic kidney disease: a promising alternative. *Am. J. Kidney Dis.* **27**, 224–233 (1996).

34. Sulikowski, T. *et al.* Laparoscopic removal of renal cysts in patients with ADPKD as an alternative method of treatment and patient preparation for kidney transplantation: preliminary results. *Transplant. Proc.* **38**, 23–27 (2006).
